# The Sysmex CS-5100 coagulation analyzer offers comparable analytical performance and excellent throughput capabilities

**DOI:** 10.1016/j.plabm.2016.09.002

**Published:** 2016-09-21

**Authors:** T. Flieder, T. Gripp, C. Knabbe, I. Birschmann

**Affiliations:** Institute for Laboratory and Transfusion Medicine, Heart and Diabetes Center, Ruhr University Bochum, Georgstrasse 11, 32545 Bad Oeynhausen, Germany

**Keywords:** Coagulation analyzer, CS-5100, Evaluation, Automation

## Abstract

**Objectives:**

This study compared the new high-volume blood coagulation analyzer Sysmex CS-5100 System™ (Siemens Healthcare Diagnostics, Erlangen, Germany) to the mid-volume blood coagulation analyzer Sysmex CS-2000*i* System™ (Siemens) for analytical performance. Additionally, the operational performance of the Sysmex CS-5100 System was compared with the blood coagulation analyzer ACL TOP 700 (Instrumentation Laboratory, Werfen Group, Kirchheim bei Munchen, Germany).

**Materials and methods:**

We compared the Sysmex CS-5100 to the Sysmex CS-2000*i* and the ACL TOP analyzer for routine coagulation, chromogenic and immunological assays. Imprecision studies were performed for the Sysmex CS-5100 and Sysmex CS-2000*i* systems. A throughput and STAT analysis comparison of the CS-5100 and the ACL TOP was performed. A stress test was performed to characterize the robustness and the error rate of the CS-5100. We also performed correlation analysis between the CS-5100 and the CS-2000*i* or the ACL TOP in the measurement of patients’ samples.

**Results:**

The inter-assay precision using the CS systems was impressive (inter-assay CV generally <3.5%) and the correlation between the two Sysmex analyzers was excellent. In the throughput study, the CS-5100 completed the measurement of 100 samples (210 results) in less than 49 min.

**Conclusions:**

Our results demonstrated that the CS-5100 is a robust high-throughput analyzer, well-suited for coagulation laboratories.

## Highlights

• We show a comparison between the CS-5100, CS-2000i and ACL TOP 700.
• CS-5100 is a high-volume coagulation analyzer with excellent inter-assay precision.
• The system has a considerable high throughput and a good software usability.
• The software was simple, intuitive to use and offers various monitoring possibilities.

## 1 Introduction

Current requirements in the hemostasis laboratory include optimal diagnostic performance, improved quality management, and efficient work-flow. The high-volume blood coagulation analyzer Sysmex CS-5100 System™ (Siemens Healthcare Diagnostics, Erlangen, Germany) is fully automated and has been developed for routine coagulation diagnostics in large hospitals.

The investigation of hemostasis has always been an important aspect of laboratory diagnostics. With the growing number of samples which a laboratory must handle, a fast, precise and robust analyzer is essential. Furthermore, any new analyzer should require minimal operational and maintenance time, and have a small sample and dead volume in order to analyze pediatric samples.

In modern laboratories most analyzers used for clinical chemistry, hematology and hemostaseology are automated for shorter turn-around-time (TAT) (especially for samples from the emergency room and intensive care unit) and to minimize the personnel needed to operate them Any new coagulation analyzer must be compatible with such systems for measuring the standard parameters (PT, PTT, fibrinogen, antithrombin, d-dimer). In particular, in laboratories associated with blood transfusion services, the precise measurement of single-factor activity is necessary to obtain all the official licenses required. The system should also perform more specific assays e.g. Protein C, Protein S, von Willebrand assays.

The operation of the analyzer and the software must be simple and intuitive because the technical staff must be able to work with the various analyzers (e.g. clinical chemistry and hemostaseology) concurrently.

## 2 Materials and methods

### 2.1 Sample collection and preparation

Leftover citrated plasma from patient samples was used to evaluate the system. Blood was collected in 2.9 mL tubes containing 290 µL sodium citrate (100 mmol/L) (KABE Labortechnik GmbH, Nümbrecht, Germany). Blood was centrifuged for 10 min at 3000×*g* for prothrombin time (PT), partial thromboplastin time (PTT), antithrombin (AT), fibrinogen (Fib) and D-dimer. For specific hemostaseological tests (factor II (FII), factor V (FV), factor VII (FVII), factor VIII (FVIII), factor IX (FIX), factor X (FX), factor XI (FXI), factor (FXII), factor XIII (FXIII), protein C (PC), von Willebrand factor antigen (VWF Ag), von Willebrand factor activity (VWF Ac), activated protein C-resistance (APC-resistance)) plasma was again centrifuged at 3000×*g* for 10 min (room temperature), frozen at −20 °C for a maximum of one week and analyzed in batches.

### 2.2 The analyzer

The Sysmex CS-5100 System is a fully automated, computer-interfaced coagulation analyzer intended for in vitro diagnostic use. The analyzer has 36 incubation wells and 20 measurement channels. The Sysmex system is able to perform clotting, chromogenic and immunological tests. The absorbance measurement unit uses a halogen lamp with 340, 405, 575, 660, and 800 nm wavelengths.

The analyzer can hold 10 racks with 10 samples per rack, with capability to mix primary tubes from different manufacturers on the same rack at the same time. It has an additional cap-piercing sample arm, which processes capped and uncapped sample tubes. Up to 40 reagents can be stored on board using the rack system, which are all cooled at 10 °C ±2 °C. Five extra positions at ambient temperature are dedicated for buffer or rinse solution. Additionally, there are five STAT positions which can be loaded continuously; the analyzer will immediately start measuring the samples. Samples, controls and reagents are identified by barcode readers. As Control plasma can be included in the reagent area, so measurement of controls can be performed at any time, for instance when a new vial of reagent is used. It is also possible to run the control from a 4 mL cup loaded on a sample rack to avoid reducing the number of reagent positions available.

During measurement, it is possible to load cuvettes (up to 1000) and samples continuously. If reagents need to be changed, the system will stop the run at the next feasible time.

Sophisticated software algorithms record, monitor, and check reaction kinetics to determine the correct clotting time. In addition, the system provides an overview of all loaded reagents showing the number of remaining tests and the time since the reagent was uploaded into the analyzer. Connection to the system manager is optional.

The analyzer performs an automatic hemolysis, icterus and lipemia check with a preanalytical scan of patient samples performed at two wavelengths (575 nm and 660 nm). A primary tube sample volume check identifies insufficient sample volume.

### 2.3 Reagents

The reagents and calibrators used on the CS-5100 and CS-2000*i* for this study are listed in Table 1. All assays were performed according to the manufacturers’ specifications and standard laboratory methods.

**Table 1:** Reagents and calibrators used on the CS-5100/CS-2000*i* analyzers for the comparison study.

	**Siemens Healthcare Diagnostics Products GmbH**
**Test**	**Reagent/Calibrator/Control**
PT	Thromborel® S/ PT Multi Calibrator/Control Plasma N, Control Plasma P, Dade® Ci-Trol® 2
	Dade Innovin®/ PT Multi Calibrator/Control Plasma N, Control Plasma P, Dade Ci-Trol 2
APTT	Dade Actin® FSL/ - /Control Plasma N, Dade Ci-Trol 2
	Pathromtin® SL/ - /Control Plasma N, Dade Ci-Trol 2
Fib	Dade Thrombin-Reagent/Standard Human Plasma/ Control Plasma N, Control Plasma P
FII/ FV/ FVII/ FX	Dade Innovin/ Standard Human Plasma/Control Plasma N, Control Plasma P
FVIII/ FIX/ FXI/ FXII	Dade Actin FSL/Standard Human Plasma/Control Plasma N, Control Plasma P
F XIII	Berichrom® FXIII/Standard Human Plasma/Control Plasma N, Control Plasma P
AT	INNOVANCE® Antithrombin/Standard Human Plasma/Control Plasma N, Control Plasma P
D-Dimer	INNOVANCE D-Dimer/INNOVANCE D-Dimer Cal/INNOVANCE D-Dimer Control 1 + 2
VWF Ac	INNOVANCE VWF Ac/Standard Human Plasma/ Control Plasma N, Control Plasma P
VWF Ag	von Willebrand Antigen/ Standard Human Plasma/ Control Plasma N, Control Plasma P
TT	Test Thrombin Reagent/ - /Control Plasma N, Control Plasma P
PC	Protein C Reagent/Standard Human Plasma/ Control Plasma N, Control Plasma P
APC	ProC® Ac R/ - /Control Plasma N, ProC Control Plasma
	**Instrumental Laboratory**
**Test**	**Reagent/Calibrator/Control**
PT	HemosIL Recombi PlasTin 2 G/HemosIL Calibration Plasma/HemosIL Normal Control, HemosIL Low Abnormal Control
APTT	HemosIL SynthASil/ - /HemosIL Normal Control, HemosIL Low Abnormal Control
	APTT-SP (liquid)/ - /HemosIL Normal Control, HemosIL Low Abnormal Control
Fib	HemosIL Q.F.A. Thrombin (Bovine)/HemosIL Calibration Plasma/HemosIL Normal Control, HemosIL Low Abnormal Control
FII/ FV/ FVII/ FX	HemosIL Factor II/ V/ VII/ X deficient plasma/HemosIL Calibration Plasma/HemosIL Normal Control, HemosIL Special Test Control Level 2
FVIII/ FIX/ FXI/ FXII	HemosIL Factor VIII/ IX/ XI/ XII deficient plasma/HemosIL Calibration Plasma/HemosIL Normal Control, HemosIL Special Test Control Level 2
F XIII	HemosIL Factor XIII Antigen/ HemosIL Calibration Plasma/HemosIL Normal Control, HemosIL Special Test Control Level 2
AT	HemosIL Antithrombin/ HemosIL Calibration Plasma/HemosIL Normal Control, HemosIL Low Abnormal Control
D-Dimer	HemosIL D-Dimer HS 500/ HemosIL D-Dimer HS 500 Calibrator/HemosIL D-Dimer HS 500 Controls
VWF Ac	HemosIL VWF Ac/HemosIL Calibration Plasma/HemosIL Normal Control, HemosIL Special Test Control Level 2
VWF Ag	HemosIL VWF Ag/HemosIL Calibration Plasma/HemosIL Normal Control, HemosIL Special Test Control Level 2
TT	HemosIL Thrombinzeit/ - /HemosIL Normal Control, HemosIL Low Abnormal Control
PC	HemosIL ProC/ HemosIL Calibration Plasma/ HemosIL Normal Control, HemosIL Special Test Control Level 2
APC	HemosIL Factor V Leiden/ - /HemosIL APC Control Plasma Level 1 + 2

PT: prothrombin time; APTT: activated partial thromboplastin time; Fib: fibrinogen, FII: factor II; FV: factor V; FVII factor VII; FVIII: factor VIII; FIX: factor IX; FX: factor X, FXI: factor XI; FXII: factor FXII; FXIII: factor XIII; PC: protein C; VWF Ag: von Willebrand factor antigen; VWF Ac: von Willebrand factor activity; APC-resistance: activated protein C-resistance.

### 2.4 Methods

The study was designed in order to evaluate the Sysmex CS-5100 in terms of hardware/software quality and in routine use. Therefore, patient samples were measured with both the CS-5100 and CS-2000*i* as well as the ACL TOP. Several aspects of the study, e.g. test samples and storage were performed based on the CLSI EP9-A2 guidelines (CLSI, Wayne, PA, USA), which we followed. After independently training two technicians, a robustness test of both Sysmex analyzers was performed over five weeks. During this period, an average of 128 samples a day (range 36–186) and a total of 3093 samples was analyzed. The same procedure was performed by the second technician for two more weeks, analyzing an average of 130 (range 67–243) samples per day and 1684 samples in total.

Imprecision was determined using the following commercial quality control materials at different concentration levels: Control Plasma N (CPN), Control Plasma P (CPP), Dade Ci-Trol 2 (Citrol 2), INNOVANCE D-Dimer Control 1, INNOVANCE D-Dimer Control 2 (Table 1). All control preparations were obtained from Siemens Healthcare Diagnostics Products GmbH (Marburg, Germany). The coefficient of variation (CV) for each material was calculated based on the daily results of measured samples.

A stress test was performed over three days. On day one the CS-5100 was used for nonstop measurement over five hours with the analysis of routine tests and a small number of specific hemostaseological ones. STAT samples were inserted randomly in the CS-5100 during the stress test. During the test, under filled bottles were used to induce a change of reagent vials. As primary tubes, pediatric tubes, and sample cups can be loaded onto the same rack, this was additionally tested under stress conditions. One the second day the analyzer was continuously loaded with samples over five hours with routine preparation of the analyzer. The throughput of the CS-5100 and ACL TOP was evaluated using the same 100 routine samples with request for PT (*n*=100), PTT (*n*=80), AT (*n*=20) and d-dimer (*n*=10). STAT capability was determined by the processing time of two STAT samples for the same four assays. Finally, on day three the barcode reader was tested using unclear barcodes, e.g. wet or damaged barcodes, on various tubes. A 24-h test was also conducted in order to simulate a routine day including reagent changes, maintenance and a shutdown of the system during which time samples were continuously delivered for measurement.

### 2.5 Statistical evaluation

The method comparison study data were evaluated by Siemens Healthcare Diagnostics Products GmbH using the statistical software SAS V9.1. Passing-Bablok regression analysis as well as Bland-Altman analysis were performed. For Passing Bablok regression we deemed an acceptable comparison to be a slope of 1.0±0,1 and a correlation coefficient *r*>0,95.

## 3 Results

### 3.1 Inter-assay precision

As shown in Table 2A, the inter-assay CVs (PT, PTT, Fib, AT) were below 3.51% for the parameters measured in controls (normal and pathological range) using the CS-5100, consistent with previous studies [1], [2]. The same was true for the CS-2000*i*, with inter-assay CVs below 5.1%. The CV for specific coagulation parameters such as FVIII, FXIII, VWF Ag, VWF Ac was also calculated when a minimum of 6 analyses of controls had been performed (Table 2B). Exceptionally, D-dimer measurement showed a higher CV for the pathological control (11.41%) whereas the normal control had a lower CV of 4.26%.

**Table 2A:** Inter-assay precision of the CS-5100 and CS-2000*i* analyzers for routine tests.

				**CS5100**			**CS2000*i***	
**Test**	**Control**	**Unit**	***n***	**Mean±SD**	**CV (%)**	***n***	**Mean±SD**	**CV (%)**
PT (Dade INNOVIN)	N	%	28	85.5±2.17	2.54	25	86±2.57	2.99
PT (Dade INNOVIN)	P	%	25	36.4±0.54	1.47	28	36.3±0.69	1.89
INR	N		28	1.07±0.01	0.92	25	1.07±0.02	1.45
INR	P		25	1.81±0.03	1.4	28	1.81±0.03	1.65
PT (Thromborel S)	N	%	32	80.8±2.83	3.51	33	87.2±4.14	4.75
PT (Thromborel S)	P	%	33	40.5±1.18	2.91	32	41.3±2.11	5.1
INR	N		32	1.11±0.02	1.99	33	1.07±0.03	2.65
INR	P		33	1.80±0.05	2.61	32	1.77±0.08	4.52
APTT (Dade Actin FSL)	N	s	33	27.1±0.17	0.63	33	27±0.26	0.95
APTT (Dade Actin FSL)	C2	s	33	48.7±0.66	1.35	33	48.7±0.67	1.38
APTT (Pathromtin SL)	N	s	32	31.5±0.85	2.7	32	31.9±0.39	1.23
APTT (Pathromtin SL)	C2	s	32	62.4±0.65	1.04	32	62.7±1.01	1.61
Fibrinogen	N	g/L	54	2.43±0.06	2.29	53	2.36±0.09	3.64
Fibrinogen	P	g/L	53	0.81±0.02	2.15	54	0.79±0.03	3.34
Antithrombin	N	%	50	94.5±2.15	2.28	48	95.4±3.14	3.29
Antithrombin	P	%	48	31.9±1.01	3.15	50	33.3±1.44	4.33
INNOVANCE D-Dimer	D1	mg/L	21	0.35±0.01	4.26	21	0.32±0.03	10.74
INNOVANCE D-Dimer	D2	mg/L	23	2.58±0.29	11.41	22	2.55±0.2	10.43

Abbreviations as in Table 1. N=Control Plasma N; P=Control Plasma P; C2=Dade Citrol 2, D1=D-Dimer control 1; D2=D-Dimer control 2. SD = standard deviation, CV=coefficient of variation.

**Table 2B:** Inter-assay precision of the CS-5100 and CS-2000*i* analyzers for special coagulation parameters.

**Test**	**Control**	**Unit**	***n***	**CS5100**		***n***	**CS2000i**	
				**Mean±SD**	**CV (%)**		**Mean±SD**	**CV (%)**
F II	N	%	8	89.5±2.55	2.86	8	93.5±2.85	3.04
	P	%	8	31.3±0.9	2.88	8	31±0.84	2.72
F V	N	%	8	92.2±2.53	2.75	8	88.8±5.79	6.52
	P	%	8	29.1±3.98	3.98	8	29.6±2.15	7.27
F VII	N	%	6	83.5±2.92	3.5	6	86.6±4.67	5.39
	P	%	6	34.8±0.75	2.15	6	35.6±1.03	2.89
F VIII	N	%	19	87.2±3.13	3.59	19	89.6±6.22	6.94
	P	%	18	27.3±1.13	4.13	21	27.3±2.48	9.08
F IX	N	%	8	90.9±2.4	2.64	15	83.8±7.2	8.59
	P	%	8	31.7±2.18	2.18	9	28.6±2.68	9.37
F X	N	%	6	84.4±2.86	3.38	6	92.3±5.25	5.69
	P	%	6	31.5±0.97	3.08	6	31.5±1.2	3.81
F XI	N	%	6	92.6±3.46	3.74	6	101.2±3.85	3.81
	P	%	6	29.1±0.24	0.84	6	28.7±1.25	4.37
F XII	N	%	6	90±4.05	4.51	6	93±5.82	6.26
	P	%	8	32.7±3.84	11.75	6	30.1±3.1	10.33
F XIII	N	%	10	85.1±2.26	2.65	15	81.2±7.53	9.28
	P	%	12	26.0±1.56	6	13	20.7±2.76	10.89
VWF Ag med	N	%	16	109.9±2.20	2	16	114.7±2.27	1.98
	P	%	16	40.1±1.13	2.82	16	40.9±1.20	2.93
INNOVANCE VWF Ac med	N	%	14	92.6±1.95	2.11	14	97.7±6.43	6.59
	P	%	14	28.9±0.47	1.62	14	28.2±1.37	4.88
Protein C	N	%	8	95.2±7.19	7.55	8	96±7.6	7.92
	P	%	8	32.4±1.97	6.08	8	35.4±3.55	10.02
APC resistance (ratio)	N	%	8	3.16±0.13	4.17	10	3.12±0.15	4.93
	P	%	8	1.15±0.02	1.64	8	1.17±0.02	1.33

Abbreviations as in Table 1. N= Control Plasma N; P = Control Plasma P. SD = standard deviation, CV = coefficient of variation.

### 3.2 Method comparison study

**Fig. 1:**
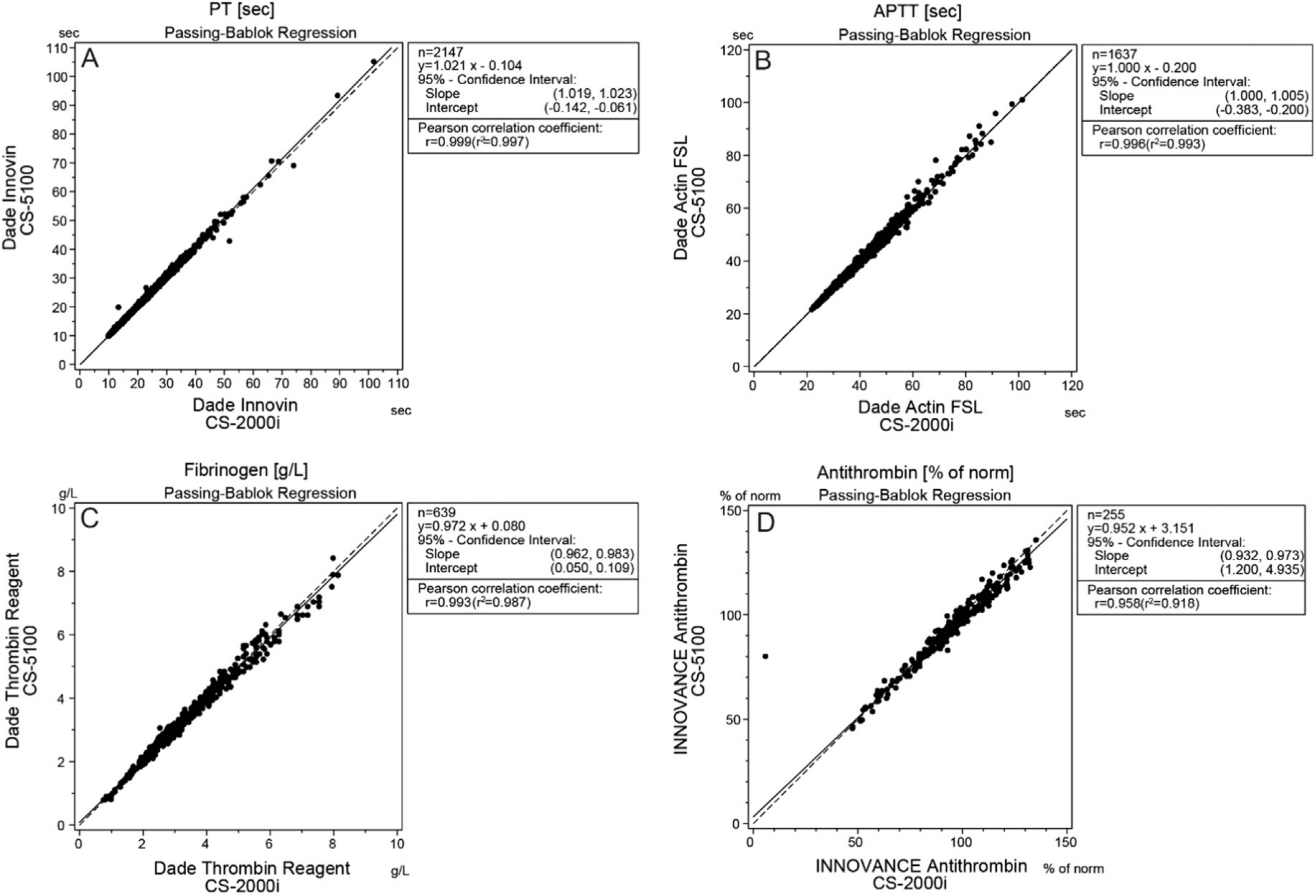
Correlation of CS-5100 to CS-2000*i* for prothrombin time (PT) (A), partial thromboplastin time (PTT) (B), fibrinogen (C) and antithrombin (D).

The results of the correlation study between the CS-5100, CS-2000*i* and ACL TOP are shown for PT, PTT, Fib and AT Fig. 1(A–D) and for PT, PTT, Fib and AT in Table 3A, Table 3B. In addition, measurements of various coagulation factors were performed according to the individual requests. However, in view of the low numbers (*n*<40), no further statistical analysis was conducted..

**Table 3A:** Correlation between the CS-5100 and the CS-2000*i* for the measurement of patients' samples.

								**CS-5100**			**CS-2000*i***		
**Test**	**Unit**	***n***	**Absolute difference |*y*–*x*| (Mean)**	**Slope**	**Intercept**	***r***	***r***^**2**^	**Range**	**Mean**	**Median**	**Range**	**Mean**	**Median**
PT (Dade Innovin)	%	2220	1.41	0.995	+0.035	0.997	0.995	7.7−137.4	59.8	54.5	5.6−42.2	60.1	55.1
PT (Dade Innovin)	s	2147	0.36	1.021	−0.104	0.999	0.997	9.9−105.2	19.0	15.3	9.8−101.8	18.7	15.1
INR		2220	0.021	1.000	+0.000	0.999	0.998	0.86− 6.29	1.65	1.34	0.86−6.73	1.65	1.33
PT (Thromborel S)	%	1313	2.56	0.921	+2.349	0.998	0.996	11.3−132.3	55.8	54.4	11.6−136.2	58	56.4
PT (Thromborel S)	s	1313	0.41	0.992	−0.096	0.998	0.996	10.3 −66.9	19.6	16.4	10.6−65.3	20.0	16.7
INR		1313	0.042	0.98	+0.053	0.998	0.996	0.87−6.32	1.73	1.42	0.85−6.29	1.72	1.4
APTT (Dade Actin FSL)	s	1637	0.61	1.000	−0.200	0.996	0.993	21.7−101.1	36.6	34.3	21.9−101.4	36.8	34.5
APTT (Pathromtin SL)	s	1004	0.96	0.986	+0.201	0.997	0.994	21.3−151.7	48.2	44.6	21.4−171.9	48.8	45.2
Fibrinogen	g/l	639	0.107	0.972	+0.080	0.993	0.987	0.79−8.42	3.42	3.24	0.75−8.14	3.43	3.24
Antithrombin	%	255	2.98	0.952	+3.151	0.958	0.918	45.6−136.0	94.3	96.2	5.8−135.2	95.3	97.1

Abbreviations as in Table 1

**Table 3B:** Correlation between the CS-5100 and the ACL TOP for the measurement of patients’ samples.

									CS-5100			ACL TOP		
**Reagent CS-5100**	**Reagent ACL TOP**	**Unit**	***n***	**Absolute difference |*y*-*x*| (Mean)**	**Slope**	**Intercept**	**r**	***r***^**2**^	**Range**	**Mean**	**Median**	**Range**	**Mean**	**Median**
PT (Dade Innovin)	HemosIL Recombi PlasTin 2G	%	2210	5.02	0.971	−1.157	0.981	0.962	7.7−137.4	59,6	54,2	10−152	63	60
PT (Dade Innovin)	HemosIL Recombi PlasTin 2G	s	2148	1.80	0.815	+2.304	0.983	0.966	9.9−105.2	19.0	15.3	8.6−98.9	20.3	16.3
INR			2221	0.122	0.870	+0.133	0.984	0.968	0.86−6.29	1,65	1,34	0.77−6.43	1.73	1.41
PT (Thromborel S)	HemosIL Recombi PlasTin 2G	%	1313	12.18	0.772	+3.776	0.978	0.957	11.3−132.3	55.8	54.4	8−181	68	68
PT (Thromborel S)	HemosIL Recombi PlasTin 2G	s	1313	2.28	0.889	+2.978	0.960	0.922	10.3−66.9	19.6	16.4	8.1−99.6	19.1	14.8
INR			1313	0.173	1.000	+0.110	0.964	0.929	0.87−6.32	1.73	1.42	0.72−8.14	1.64	1.30
APTT (Dade Actin FSL)	HemosIL SynthASil	s	1637	4.83	0.920	−0.012	0.871	0.759	21.7−101.1	36,6	34,3	20.7−160.7	39.8	36.8
APTT (Pathromtin SL)	HemosIL SynthASil	s	1005	8.90	1.565	−14.024	0.899	0.808	21.3−151.7	48.2	44.5	18.3−126.5	39.8	36.3
Fibrinogen	HemosIL Q.F.A. Thrombin (Bovine)	g/l	641	0.397	1.064	+0.189	0.975	0.95	0.79−8.47	3.43	3.24	0.72−8.16	3.06	2.79
Antithrombin	HemosIL Antithrombin	%	256	5.38	0.886	+12.279	0.941	0.886	45.6−136.0	94.4	96.3	24−144	93	93

Abbreviations as in Table 1

**Fig. 2:**
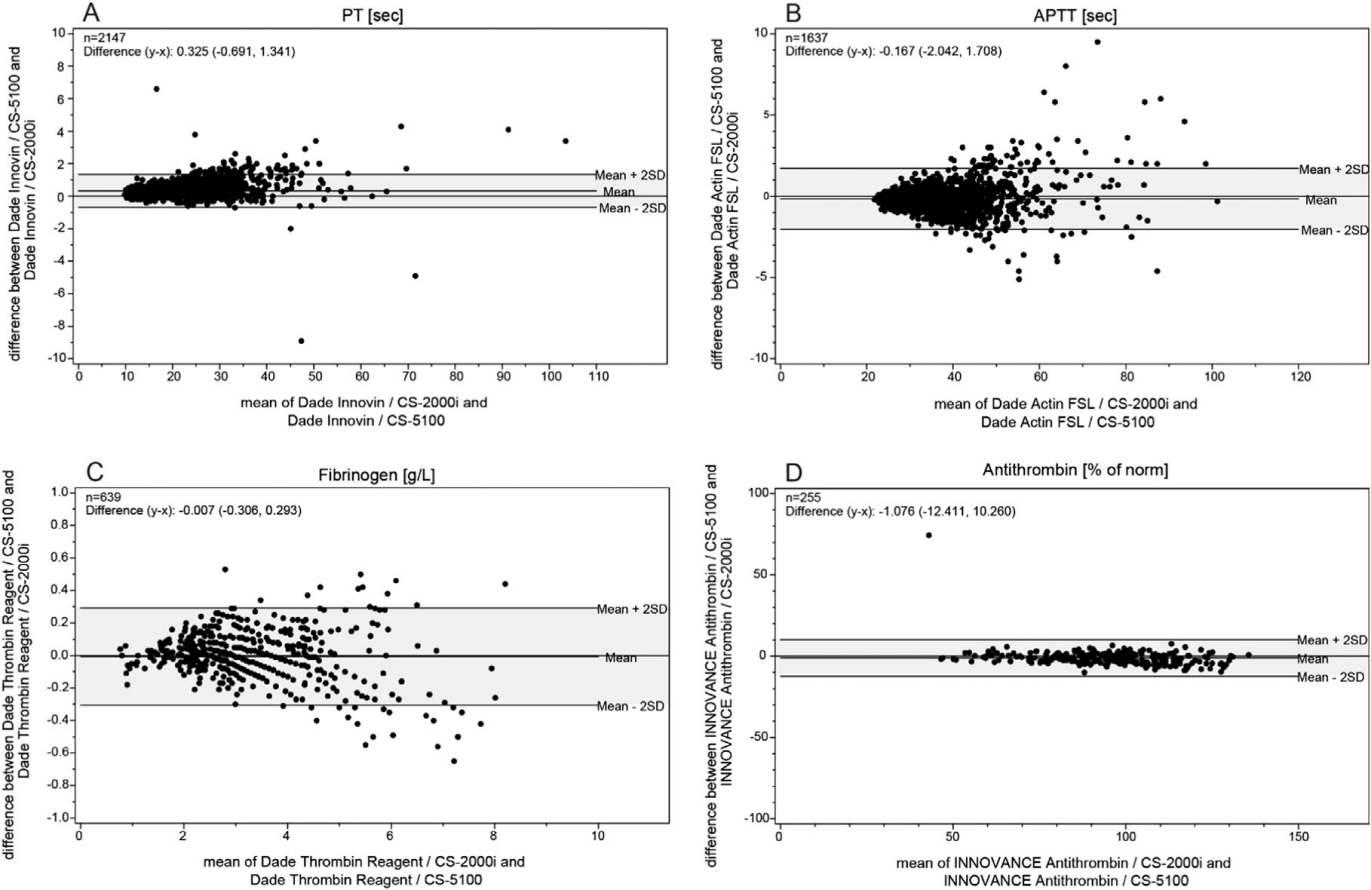
Bland-Altman plots for prothrombin time (PT) (A), partial thromboplastin time (PTT) (B), fibrinogen (C) and antithrombin (AT) (D).

The agreement between results obtained from different analyzers is demonstrated for PT, PTT, Fib and AT in difference-plots according to Bland and Altman (Fig. 2A–D). A complete list of all results is shown in Table 4..

**Table 4:** Comparison of analyzers using Bland-Altman difference plots–summary of differences.

**Test/unit**	**Reagent**	**CS-5100/CS-2000*****i***		**CS-5100/ACL TOP**	
		***n***	**Mean of differences (*x*–y) ±2SD**	***n***	**Mean of differences (*x*–y) ±2SD**
PT [s]	Dade Innovin	2147	0.325 (−0.691, 1.341)	2148	−1.332 (−5.933, 3.268)
	Thromborel	1313	−0.324 (−1.392, 0.744)	1313	0.573(−6.765, 7.911)
PT [%]	Dade Innovin	2220	−0.266 (−4.852, 4.320)	2210	−3.209 (−15.892, 9.474)
	Thromborel	1313	−2.155 (−7.716, 3.405)	1313	−11.666 (−29.791, 6.459)
INR	Dade Innovin	2220	0.006 (−0.072, 0.083)	2221	−0.081 (−0.400, 0.239)
	Thromborel	1313	0.013 (−0.104, 0.129)	1313	0.088 (−0.405, 0.580)
PTT [s]	Actin FSL	1637	−0.167 (−2.042, 1.708)	1637	−3.206 (−16.456, 10.043)
	Pathromtin SL	1004	−0.643 (−4.031, 2,744)	1005	8.327 (−9.384, 26.038)
Fibrinogen	Dade Thrombin Reagent	639	−0.007 (−0.306, 0.293)	641	0.374 (−0.188, 0.936)
Antithrombin	INNOVANCE Antithrombin	255	−1.076 (−12.411, 10.260)	256	1.831 (−11.906, 15.567)

The small differences between the two analyzers are demonstrated using Bland-Altman plots which show that consideration of the differences is not essential for making clinical decisions. The Bland-Altman plot of PTT using Actin FSL reagent, for example, showed a mean time difference of −0.167 s with a ±2SD range of −2.042 to 1.708 s. This is clearly a strong indication of the good correlation between the two analyzers where no clinically relevant differences could be observed. Furthermore, the plots reveal no systematic bias and, as shown in Fig. 2A–D, there are also no deviations between the means of the two systems. However, although the results obtained from comparison of the two CS systems showed excellent correlation, small differences could be detected when we compared the ACL TOP to the CS-5100.

### 3.3 Interference

Analytical interference with spectrophotometric methods occurs with hemolysis, bilirubin and lipids with laboratory assays. As a consequence, the altered results may lead to inappropriate further tests, incorrect interpretation, incorrect diagnosis and potentially needless intervention and unfavorable outcome for patients [3]. The hemolysis, icterus and lipemia (HIL) check detects these potential interferences with using a photometric absorbance check on patient samples at relevant wavelengths.

To verify the ability of the CS-5100 to detect hemolytic and lipemic samples, two panels consisting of 37 and 4 samples, respectively, were identified by visual assessment and were quantified for free hemoglobin and triglycerides. As most of the hemostatic assays were not affected significantly by bilirubin, no further analyses of icteric samples were carried out in the study.

From the hemolytic sample panel 33 samples were confirmed by measurement of free hemoglobin >50 mg/dL. This concentration has been shown to influence various spectrophotometric assays. The CS-5100 flagged 32 samples of these samples correctly (97%). Three samples were flagged but had free hemoglobin concentrations below 50 mg/dL (31 mg/dL, 39 mg/dL and 43 mg/dL) and one sample with a free hemoglobin concentration of 60 mg/dL was not flagged by the CS-5100.

Interference of lipids on hemostatic assays is supposed to be significant at >150 mg/dL triglyceride [4]. All four selected lipemic samples, were confirmed by measurement of triglycerides as >400 mg/dL. The CS-5100 flagged all samples but one. The concentration of triglyceride in this sample was extremely high (943 mg/dL). This discrepancy may be caused by the fact that lipids vary greatly in size and composition, and thus true triglyceride concentrations do not always correlate with the results from photometric interference detection.

### 3.4 Stress test

On the first day of the stress test the analyzer had no problems with vial changes following used-up reagents or sample aspiration with different primary tubes and sample cups. STAT samples were given high priority when they were started. No major errors were observed during the first day instead of flags because of used-up reagent or quality control results outside the target intervals.

On the second day of the stress test, throughput of the CS-5100 was compared to the ACL TOP and determined by the processing times for testing 100 samples with a mixed assay profile (PT, PTT, AT and D-dimer; 210 results). The results of the throughput study are presented in Table 5. The first result was shown by the ACL TOP analyzer after 6 min 10 s, whereas the CS-5100 finished the first sample after 8 min 28 s. For the measurement of all 100 samples, the CS-5100 was 38% faster (48 min 51 s) than the ACL TOP (67 min 24 s). STAT capability was determined by the processing time of two STAT samples for the same four assays. Although the CS-5100 required two minutes more to report the first result, it completed the full job list (218 results) 18 min ahead of the ACL TOP (27% faster). STAT processing was more than twice as fast with the CS-5100 compared to ACL TOP (55% faster).

**Table 5:** Throughput study of the CS-5100 analyzer compared to the current laboratory analyzer ACL TOP.

**Device**	**Assays (% of samples analyzed for this parameter)**	**Result of the first sample (min:sec)**	**Results after 30 min (*n*=)**	**Results after 60 min (*n*=)**	**Result of 100 samples (min:sec)**	**Required time for 2 STAT samples with all 4 assays (min)**
CS- 5100	PT with Thromborel S (100%) APTT with Pathromtin SL (80%) AT with INNOVANCE AT (20%) D-Dimer with INNOVANCE D-Dimer (10%)	08:28	PT: 54 APTT: 44 AT: 12 D-Dimer: 6	PT: 123 APTT: 99 AT: 25 D-Dimer: 13	48:51	09:01
ACL TOP	PT with RecombiPlasTin 2G (100%) APTT with SynthASil (80%) AT with Liquid Antithombin (20%) D-Dimer with D-Dimer HS 500 (10%)	06:10	PT: 40 APTT: 32 AT: 8 D-Dimer: 4	PT: 87 APTT: 69 AT: 17 D-Dimer: 9	67:24	19:58

The barcode reader showed very good functionality and was able to read e.g. distorted and crumpled barcodes. The problems in reading the barcode occurred when the barcode was very wet or when it was overwritten with a pencil.

## 4 Discussion

This study compared the high-volume blood coagulation analyzer Sysmex CS-5100 System to the mid-volume blood coagulation analyzer Sysmex CS-2000*i* System for analytical performance. Additionally, these data were compared to the high-volume blood coagulation analyzer ACL TOP for operational performance.

The analytical performance for several assays on the CS-5100 was compared to that of the CS-2000*i* using the same Siemens reagents on both systems. The agreement between the two CS-systems was excellent. We show here for the first time a comparison of the CS-5100 and the CS-2000*i* or the ACL TOP, which included evaluation of the analytical and technical performance. The inter-assay CVs obtained were excellent at all concentrations measured. The results are similar to those obtained in other studies with fully automated coagulation analyzers ACL TOP [5], [6], Sysmex CA 6000 System [7] and Sysmex CA-7000 System [8].

As stated by the manufacturer, the CS-5100 has a higher throughput than the CS-2000*i*. Compared to the ACL TOP the CS-5100 showed considerably higher throughput while maintaining similar accuracy and imprecision.

For emergency (STAT) samples, a specific sampling position can be used which guarantees preferential treatment of the sample and fast results during routine operations.

During the study, the CS-5100 stopped once while it was measuring a high concentration made up of FVIII, von Willebrand factor antigen and INNOVANCE VWF Ac in a single blood sample taken from one patient. The cause of the stop was the fact that the CS-5100 had taken an insufficient volume of plasma aliquot from the total plasma sample. The device was thus unable to carry out a second measurement. Remarkably, this was the only system failure during the entire study period of 4 months. Since then, the error has been corrected by updating the software. Furthermore, no technical support was needed on site during the evaluation, showing that the system is robust.

The analyzer enables real-time monitoring of each sample status on-board, the status of the on-board time of each reagent and gives an overview of the number of tests that can be performed with this reagent volume. The software was extremely simple and intuitive to use.

Performance under stress conditions (running the system under simulated/provoked stress conditions) and usability during high throughput analysis were very good (data not shown).

Using the HIL check, the analyzer provides the routine technician with a fast and objective preanalytical sample check. However, some limitations were evident in an extremely lipemic sample.

Overall, the CS-5100 is excellent in terms of user needs and requirements under routine operating conditions.

The Sysmex CS-2000*i* System is not available for sale in the USA. Product availability varies by country.

Actin, Berichrom, Ci-Trol, Dade, INNOVANCE, Innovin, Pathromtin, ProC and Thromborel are trademarks of Siemens Healthcare Diagnostics Inc. or its affiliates.

Sysmex is a trademark of Sysmex Corporation.

## Conflict of interests

This study was funded by Siemens Healthcare Diagnostics Products GmbH.

IB has acted as paid speaker in the past for CSL Behring GmbH, Bristol-Myers Squibb GmbH & Co. KGaA and Instrumentation Laboratory GmbH and has received travel expenses from Siemens Healthcare Diagnostics Products GmbH.

TF and TG have received travel expenses from Instrumentation Laboratory GmbH.

CK declares no conflicts of interest.
